# Supplementary material for: Improved Robustness for Deep Learning-based Segmentation of Multi-Center Myocardial Perfusion MRI Datasets Using Data Adaptive Uncertainty-guided Space-time Analysis
Source: ArXiv. 2024 Aug 9:arXiv:2408.04805v1. Preprint. [Version 1] (PMC11326424)
Supplement: Supplement 1 [file NIHPP2408.04805v1-supplement-1.pdf]

SUPPLEMENTARY MATERIAL: APPENDICES

Appendix A – Additional Details for the Data Augmentation and Model Training Processes

Data augmentation was performed to boost the training set by $\approx$40-fold using random rotations (±60°), shear (±10°), translations (±2 pixels), uniform scaling (randomly selected scaling factor in the [0.8, 1.2] range), flat-field correction (using the "imflatfield" routine in Matlab, Mathworks, Natick, MA) with 50% probability ($\sigma \in [0, 5]$), and gamma correction with 50% probability ($\gamma \in [0.5, 1.5]$) applied to each perfusion image series in the training dataset. The augmented training dataset contained 137,160 space-time patches.

For training of the DNN models in this study, we used a cross-entropy loss with the Adam optimizer and initialized the network parameters using the He initializer (50) and employed a batch size of 128. A linear learning rate drop was utilized at every two epochs with a drop factor of 0.5 with an initial learning rate of 5×10^{-4}. We ran the training process for a maximum of 15 epochs and employed "early stopping" if the myocardial Dice score on the validation set was not improving over 5 epochs. It should be noted that, compared to image-level models, patch-level models tend to converge in fewer epochs thanks to the multi-fold increase in the size of the training dataset.

Appendix B – Additional Details for Patch-level DNN Training and Analysis

To further optimize the patch-level analysis technique, we experimented with different patch sizes and the spatial sliding-window stride. Among a set of three candidate patch sizes (16×16, 32×32, 64×64), a spatial patch size of 64×64 resulted in the best performance (validation dataset). The networks trained with smaller patch sizes (16×16 or 32×32) had difficulty focusing on global features (e.g., position of the septal wall relative to the location of bloodpool, etc.), resulting in inferior performance. As a result of working with a relatively large patch size 64×64, nearly all extracted patches included features from the heart ROI. Next, having selected a patch size of 64×64, we evaluated the effect of stride size in a similar fashion. We found that a relatively large stride size of 50% (i.e., 32 pixels with a patch size of 64×64) performed well. Finally, we used a much smaller stride size of 3% (2 pixels) for computing the U-maps. Using this small stride size helps to minimize the "edge effects" and potentially allows for a more accurate pixel-wise U-map since it enables a single spatiotemporal pixel location to be analyzed (segmented) many times.

As described in Fig. 3(a), patch-level segmentation results need to be combined to generate the final (full ROI) analysis. This process involves a "voting method" whereby each pixel in the final segmentation result is classified as being inside/outside of the LV myocardium (class 0 and class 1, respectively). We experimented with two voting methods to decide the class of each pixel when reconstructing full-ROI images from patches: (i) majority voting wherein a pixel is determined to belong to class 1 if at least half of the patches that it belongs to are showing a probability >0.5 for it to be a myocardial pixel, (ii) computing the mean of the softmax probabilities for a given pixel and applying a binarization (0.5 threshold). The results in the validation dataset did not exhibit a significant difference in terms of segmentation performance (Dice score); therefore, we proceeded with the mean of the softmax probabilities.

Appendix C – Further details on U_{pp} calculation and derivation of the U-map range

First, we provide a mathematic description of the patch combination process that is pictorially described in Fig 3(a). The binary segmentation solution, denoted by S-map, at a given spatial coordinate (x, y) is derived from the mean of the probability scores from the patches that are in $\Gamma_{(x,y)}$ followed by a binarization operation, that is:

$$\text{S-map}(x,y) = \begin{cases} 1, & if \ \frac{1}{|\Gamma_{(x,y)}|}\sum_{i=1}^{|\Gamma_{(x,y)}|} p^i \ \geq \ 0.5 \\ 0, & otherwise. \end{cases}$$

It is also worth mentioning that N_{myo}, the number of myocardial pixels in the segmentation solution, can be derived as:

$$N_{myo} = \sum_{x,y} \text{S-map}(x,y)$$

Additionally, the maximum value that a pixel can reach in the U-map is achieved when there is a maximum level of variation in the probability scores p^i corresponding to that pixel location. This "maximum level of discrepancy" between patches for a fixed pixel location (x, y) happens when half of the patches have a probability score of zero for this pixel location and the other half have a probability score of one at the same pixel location (which also implies that the mean value across all $\Gamma_{(x,y)}$ patches at (x, y) will be 0.5). Hence, the standard deviation of these softmax probabilities, which corresponds to the maximum value U-map(x, y) can assume, is:

$$\mathrm{SD_{max}} = \sqrt{\frac{\sum_i |p^i - 0.5|^2}{|\Gamma_{(x,y)}|}} = \sqrt{\frac{\frac{|\Gamma_{(x,y)}|}{2}(1-0.5)^2 + \frac{|\Gamma_{(x,y)}|}{2}(0-0.5)^2}{|\Gamma_{(x,y)}|}} = 0.5$$

On the other hand, U-map(x, y) is minimized when there is consensus regarding the rounded softmax probability score at (x, y) among all the patches that this pixel belongs to, which would result in a standard deviation of $\mathrm{SD_{min}}$ = 0. Therefore, the dynamic range of the U-map is in the [0, 0.5] range.

Appendix D - Effect of Simulated Motion-correction Error on the Uncertainty Metric

To complement our prior work on verifying the utility of U-map in interpreting patch-level segmentation of CMR perfusion datasets (24, 25), we conducted a set of simulation experiments to demonstrate that our mean per-pixel uncertainty metric (U_{pp}) is able to track the level uncertainty associated with difficulty/challenge in myocardial segmentation by varying the quality of nonrigid motion correction (MoCo) in an example stress image series. In general, the more MoCo errors we have in a test case, the more uncertain we expect the DNN-derived segmentation to be. Our simulations results, presented in the Suppl. Figure S1, are consistent with this observation (details provided in the caption).

Appendix E – Impact of improved segmentation accuracy on fully quantitative myocardial blood flow analysis

We evaluated the impact of automatic DNN-derived segmentation accuracy on fully quantitative analysis of stress perfusion CMR in two of the three test sets: inD-test and exD-1. Specifically, we compared the segment-wise MBF values obtained based on the segmentation contours from the proposed DAUGS analysis and the established approach using the Fermi-constrained deconvolution for dual-bolus stress perfusion CMR (51, 52). Landmark (RV insertion point) detection and the division of the segmentation solutions into 6-segment was automatically performed thanks to the RV segmentation ability of all trained DNNs in this work. Supplementary Fig. S2 shows the MBF results for inD-test which shows that proposed DAUGS analysis approach performed similarly to the established DNN-based analysis (R^2=0.91, p<0.0001 for both). However, on exD-1, the MBF values corresponding to the segmentations obtained from the proposed approach resulted in a stronger correlation compared to the established approach with fewer outliers (R^2=0.95 vs. R^2=0.87) and tighter 95% limits of agreement in Bland-Altman analysis (details provided in Suppl. Figure S3). These

results parallel the segmentation performance gap between two methods in terms of Dice score. In Suppl. Figure S3(c), we highlight three outliers from panel (a), which correspond to 3 myocardial segments belonging to the same perfusion image series. As can be seen from (b) and (c), in contrast to the established approach, the proposed method provides a relatively accurate segmentation which is reflected in how the MBF numbers for these 3 segments (labeled as A, B, and C) are distributed in the two plots in (b).

Appendix F – Heterogeneity of segmentation solutions in the model pool for the two external datasets

In the proposed DAUGS analysis approach, the fifty-member model pool (Fig. 1) is created by including the DNN models obtained during the training process (on the internal dataset) from different training sessions/runs (each with a different parameter initializations), or by including the intermediate checkpoints. Here, we examined the heterogeneity (diversity) of the segmentation solutions in the DNN model pool for the two external datasets by visualizing the segmentation solutions across all members of the model pool, the corresponding U-maps, and the histogram of the uncertainty metric (U_{pp} values). Specifically, we picked two external patient studies: one from exD-1 and one from exD-2.

The results are shown in Suppl. Figure S4 (caption includes additional details). As can be seen from the figure, in both examined cases, there is a high level of heterogeneity among the model pool members in terms of quality of segmentation (including failed or non-contiguous segmentation), the U-map composition, and the corresponding U_{pp} metric when there is a need to deal with "dataset shifts" that are present in the external datasets. Furthermore, there is not a particular model in the model pool that does best for all samples of the external dataset (also observed in Suppl. Fig. S4) which is consistent with the DAUGS analysis approach wherein the model selection process is data-adaptive (bottom half of Fig. 1).

Appendix G – Alternatives to U_{pp} that can be derived from U-map

The data-adaptive model selection step in the proposed DAUGS analysis technique is based on a metric of uncertainty, which is used to rank the segmentation solutions of the model pool and pick the one with the lowest uncertainty as the "best" solution. In Methods and Results, we defined and used the mean "per pixel" uncertainty metric, U_{pp}, for this purpose. However, there are many potential alternatives to U_{pp} and exploring the "optimal" design of a

mapping from the two-dimensional U-map to a scalar uncertainty metric remains as future work. Here we explored a potential alternative to U_{pp}, that is, the total energy of the U-map (without normalization by the number of myocardial pixels): $U_{tot} = ||\mathrm{U-map}||_F^2$.

We then repeated the experiments (those described in the first subsection of Results) to evaluate the segmentation performance using this alternative uncertainty metric (U_{tot} instead of U_{pp}) for all three test sets (inD-test, exD-1, and exD-2). The resulting Dice scores were: 0.896 ± 0.048 for inD-test, and 0.878 ± 0.053 for exD-1, and 0.797 ± 0.142 for exD-2. These average Dice scores are slightly lower than those obtained with U_{pp} (Fig. 4) although the difference is not statistically significant for any of the test sets. By inspecting the case by case differences (in exD-1 and exD-2), we noticed that, in challenging cases (e.g., thin apical slices) the U_{tot}-selected solutions tend to have more noncontiguous contours. An example from exD-1 is shown in Suppl. Figure S5. This improved performance can be explained by the fact that U_{pp} (unlike U_{tot}) can (to some extent) regularize the model selection process (which can be thought of as an "inverse problem" with side information) by penalizing the solutions that have very few myocardial pixels. This type of regularization in effect encodes a form of "spatial information" from the U-map into the scalar metric U_{pp}. It remains to be seen if an alternative "functional" (a general form of mapping from the 2D U-map to a scalar) that is more sophisticated than U_{pp} can be designed to further improve the segmentation performance.

Appendix H – Additional evaluation of the generalization capability of the proposed method

To further evaluate the generalization capability of the proposed DAUGS analysis method, i.e., its performance on the external datasets, we compared its performance to a new DNN that does not suffer from "dataset shifts" (13). To this end, we trained this dataset-shift-free DNN with partitioned the entire external data (combining exD-1 and exD-2) into training, validation, and testing subsets as described below:

— external training subset: n = 40 (n = 30 from exD-1; n = 10 from exD-2);

— external validation subset: n = 4 (n = 3 from exD-1; n = 1 from exD-2);

— external test subset: n = 11 (n = 7 from exD-1; n = 4 from exD-2).

During training the same augmentation setting described in Appendix A were applied and the external training subset (n = 30 from exD-1; n = 10 from exD-2) was augmented 40-fold. Similarly to the established method, we ran 5 independent training sessions and the best performing DNN was chosen based on the segmentation performance (Dice)

on the external validation subset (n = 3 from exD-1; n = 1 from exD-2). This dataset-shift-free DNN resulted in a Dice score of 0.897 ± 0.10 on the external test subset (n = 7 from exD-1; n = 4 from exD-2). On this same external test subset, the network trained on the internal dataset (with no access to the external datasets) using the DAUGS analysis approach achieved a Dice score of 0.867 ± 0.06. Although the mean Dice score for the latter network is slightly lower than the dataset-shift-free DNN (as expected) the difference was not statistically significant (p=0.3).

Supplementary Figure S1

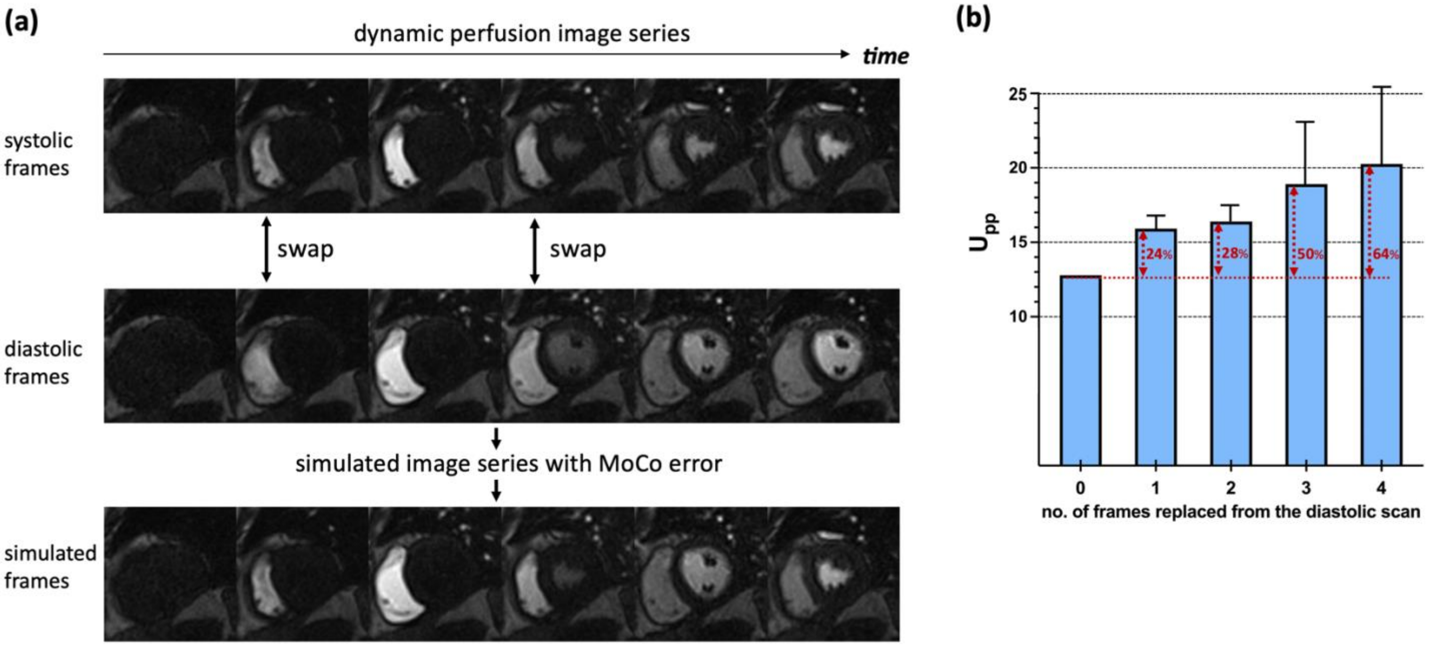

Supplementary Figure S1 (Additional File 1). Effect of simulated nonrigid motion correction error on the uncertainty quantification metric (Appendix D). To verify that our per-pixel uncertainty metric behaves as expected in presence of poor motion correction (MoCo) quality, we carried out the following two steps in an example stress perfusion CMR case: **(a)** Generating simulated perfusion image series with imperfect MoCo: as described in Panel (a), this was accomplished by replacing a small subset of systolic frames (mid slice) with diastolic frames (basal slice) from the same scan to create a simulated image series with poor MoCo quality while keeping the temporal dynamics consistent with a typical first-pass perfusion scan; specifically, a total of 30 Monte Carlo simulations were performed where f = 0,1,…,4 time frames (f = 2 in the example shown here) from the diastolic image series (basal slice) replaced the corresponding systolic frames (mid slice). **(b)** The mean per-pixel uncertainty (U_{pp} averaged over 30 Monte Carlo simulations) is plotted; as expected, the uncertainty metric increases as MoCo quality deteriorates.

Supplementary Figure S2

(a)

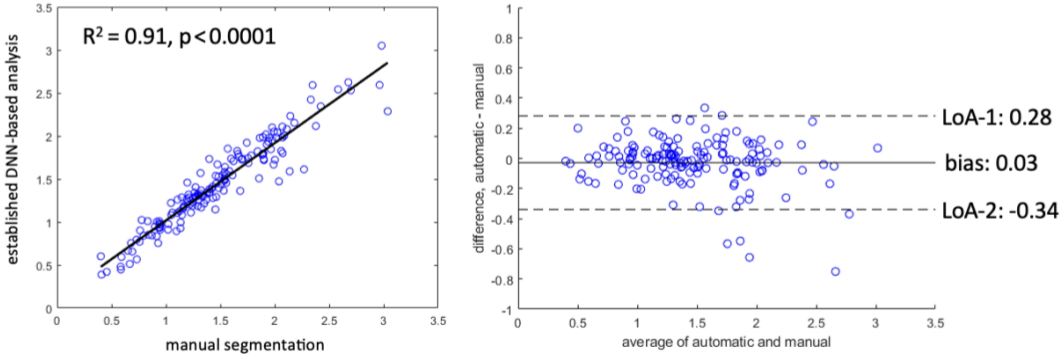

(b)

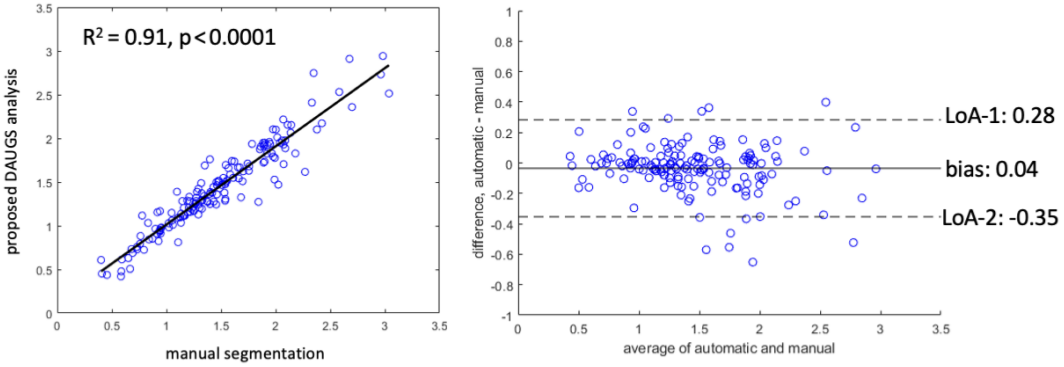

Supplementary Figure S2 (Additional File 2). Myocardial blood flow quantification results for the internal test set, inD-test (Appendix E). For further verification of the segmentation results, we quantified myocardial blood flow (in mL/g/min) using the established look-up-table (LUT)-based [Gd] Fermi-constrained deconvolution in 6-segment myocardial divisions for (a) established DNN-based analysis and (b) proposed DAUGS analysis. Both methods demonstrated strong agreement with respect to manual analysis (ground-truth MBF numbers) in terms of correlation coefficient (both approaches: R^2=0.91, p<0.0001) and the 95% limits of agreement (LoA) for Bland-Altman analysis (established approach: LoA-1: 0.28, LoA-2: -0.34, bias: 0.03; DAUGS analysis: LoA-1: 0.28, LoA-2: -0.35, bias: 0.04).

Supplementary Figure S3

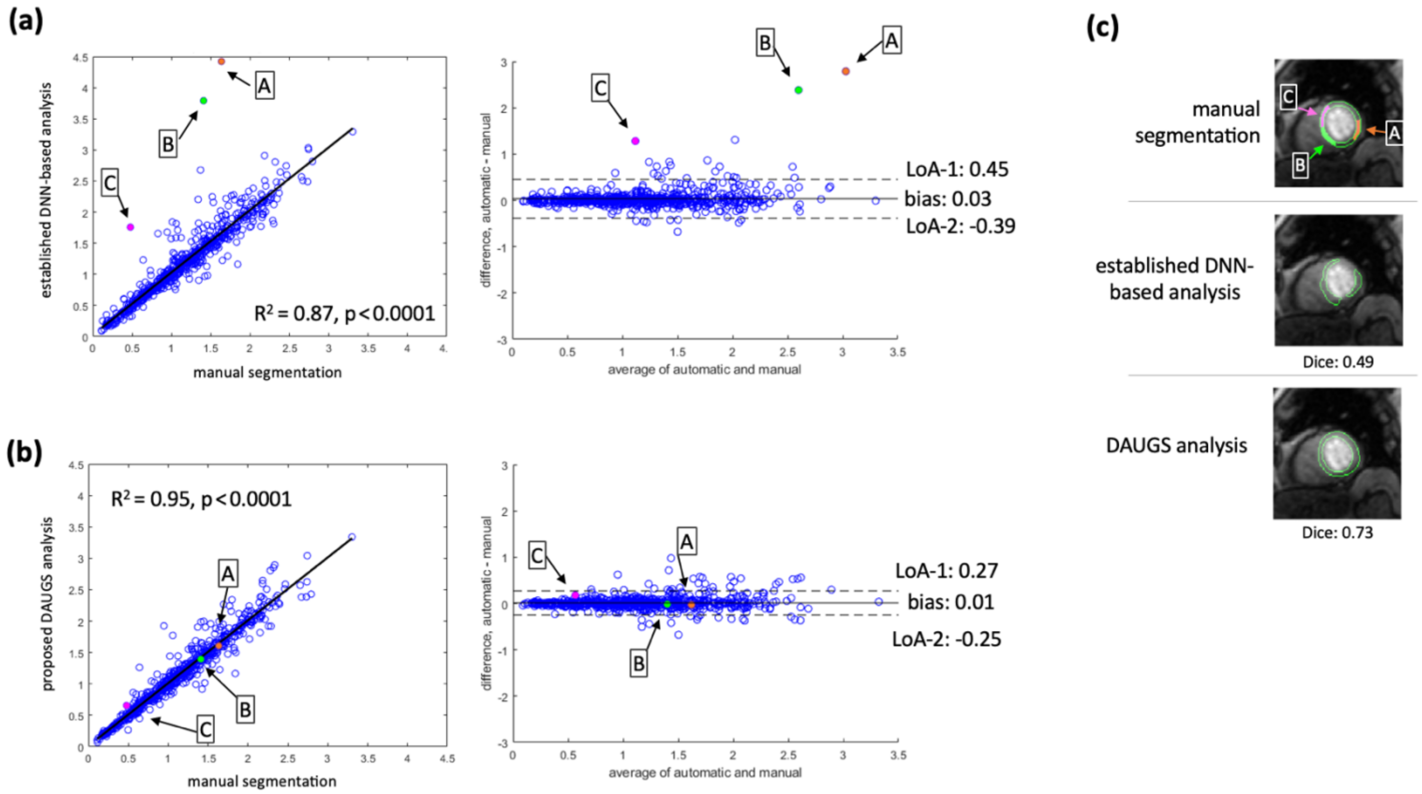

Supplementary Figure S3 (Additional File 3). Myocardial blood flow quantification results for an external test set, exD-1 (Appendix E). MBF results for exD-1 is presented for (a) established DNN-based analysis and (b) proposed DAUGS analysis. DAUGS analysis outperformed the established approach by achieving a stronger correlation (established approach: R^2=0.87, p<0.0001; DAUGS analysis: R^2=0.95, p<0.0001) and tighter limits of agreement in the Bland-Altman plots with respect to manual analysis (established approach: LoA-1: 0.45, LoA-2: -0.39, bias: 0.03; DAUGS analysis: LoA-1: 0.27, LoA-2: -0.25, bias: 0.01). **(c)** Segmentation results for three outliers (3 myocardial segments belonging to the same myocardial slice) that are highlighted as points A, B, and C in (a) and (b) as well.

Supplementary Figure S4

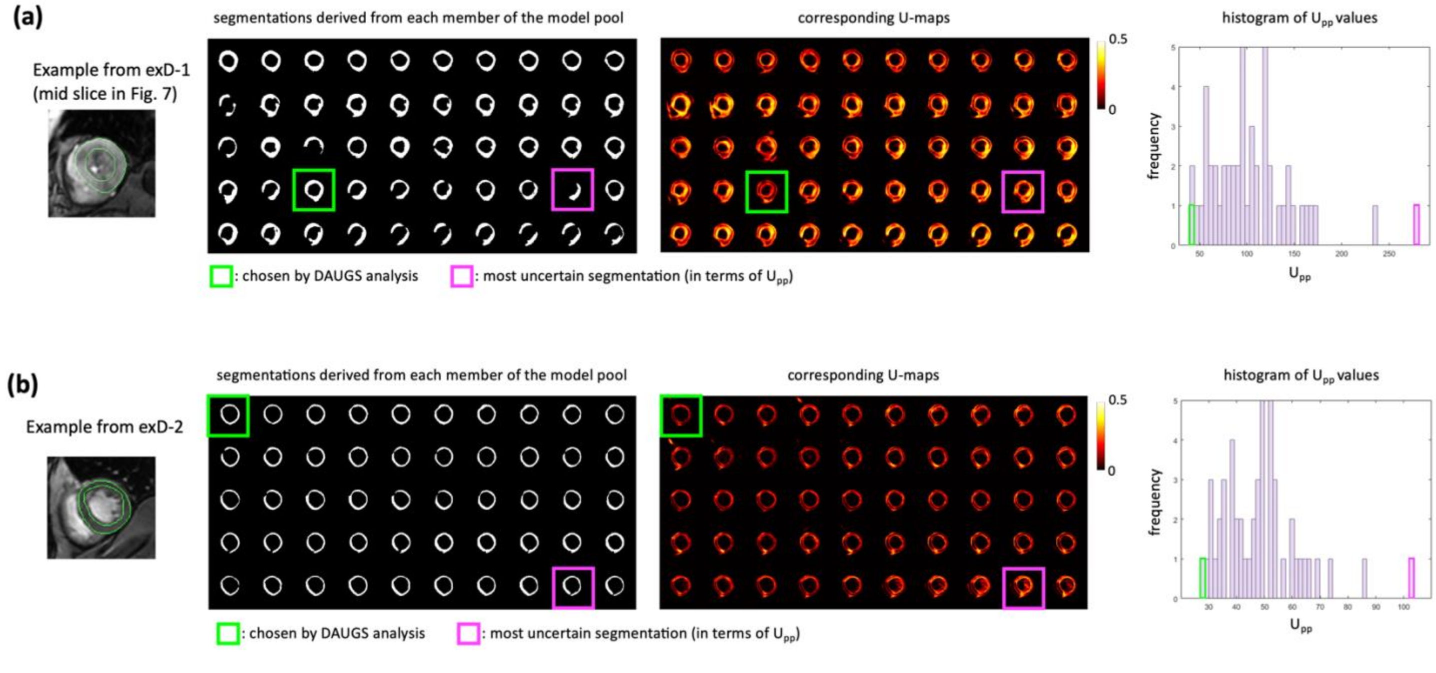

Supplementary Figure S4 (Additional File 4). Examining the heterogeneity of segmentation solutions in the DNN model pool for two example dynamic image series (Appendix F). (a) and (b) correspond to the segmentation solutions in the model pool for the proposed DAUGS analysis approach (a total of 50 DNN models as described in Fig. 1) corresponding to two external patient studies: one from exD-1 (same patient as Fig. 7) and one from exD-2. Each panel also includes the corresponding U-maps for each of the 50 segmentation solutions (middle) and show the distribution (histogram) of the uncertainty metric U_{pp} (right side). In the 5x10 matrix of segmentation solutions (and the corresponding U-maps), each row corresponds to one of the 5 training runs/sessions and each column corresponds to one of the 10 checkpoints (i.e., 10 snapshots of the working DNN model during the training process on the internal dataset). These observations support the notion that there is noticeable heterogeneity/diversity (including failed segmentations) among the 50 members of the model pool when tested on the external datasets.

Supplementary Figure S5

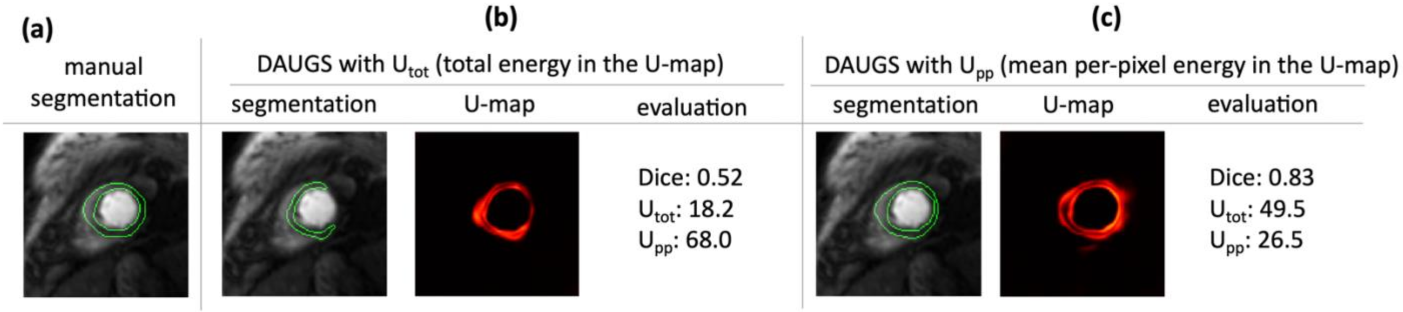

Supplementary Figure S5 (Additional File 5). Uncertainty-guided model selection with total U-map energy (U_{tot}) vs. mean per-pixel energy, U_{pp} (Appendix G). **(a)** Example apical slice from the external dataset exD-1 with manual segmentation; **(b)** DAUGS analysis results using the alternative uncertainty metric (U_{tot} instead of U_{pp}) which results in noncontiguous contours (U_{tot} is minimized for this selected solution but U_{pp} is not); **(c)** DAUGS analysis results using the proposed uncertainty metric (U_{pp}) which results in contiguous contours and a higher Dice score (U_{pp} is minimized for this selected solution but U_{tot} is not).
